# Postpartum contraceptive use and unmet need for family planning in five low-income countries

**DOI:** 10.1186/1742-4755-12-S2-S11

**Published:** 2015-06-08

**Authors:** Omrana Pasha, Shivaprasad S Goudar, Archana Patel, Ana Garces, Fabian Esamai, Elwyn Chomba, Janet L Moore, Bhalchandra S Kodkany, Sarah Saleem, Richard J Derman, Edward A Liechty, Patricia L Hibberd, K Michael Hambidge, Nancy F Krebs, Waldemar A Carlo, Elizabeth M McClure, Marion Koso-Thomas, Robert L Goldenberg

**Affiliations:** 1 Department of Community Health Sciences Aga Khan University, Karachi, Pakistan; 2 KLE University's Jawaharlal Nehru Medical College, Belgaum, Karnataka, India; 3 Lata Medical Research Foundation, Nagpur, India; 4 FANCAP, Guatemala City, Guatemala; 5 Department of Pediatrics, Moi University, Eldoret, Kenya; 6 Department of Pediatrics, University of Zambia, Lusaka, Zambia; 7 Social, Statistical and Environmental Sciences Research Triangle Institute, Durham, North Carolina, USA; 8 Department of Obstetrics and Gynecology, Christiana Care Health Services, Newark, Delaware, USA; 9 Department of Pediatrics, Indiana University School of Medicine, Indianapolis, Indiana, USA; 10 Department of Pediatrics, Massachusetts General Hospital for Children, Boston, Massachusetts, USA; 11 Department of Pediatrics, University of Colorado Health Sciences Center, Denver, Colorado, USA; 12 Department of Pediatrics, University of Alabama at Birmingham, Birmingham, Alabama, USA; 13 NICHD, Bethesda, MD, USA; 14 Department of Obstetrics and Gynecology, Columbia University, New York, New York, USA

**Keywords:** Contraception, low-middle income countries, obstetric care, family planning

## Abstract

**Background:**

During the post-partum period, most women wish to delay or prevent future pregnancies. Despite this, the unmet need for family planning up to a year after delivery is higher than at any other time. This study aims to assess fertility intention, contraceptive usage and unmet need for family planning amongst women who are six weeks postpartum, as well as to identify those at greatest risk of having an unmet need for family planning during this period.

**Methods:**

Using the NICHD Global Network for Women’s and Children’s Health Research’s multi-site, prospective, ongoing, active surveillance system to track pregnancies and births in 100 rural geographic clusters in 5 countries (India, Pakistan, Zambia, Kenya and Guatemala), we assessed fertility intention and contraceptive usage at day 42 post-partum.

**Results:**

We gathered data on 36,687 women in the post-partum period. Less than 5% of these women wished to have another pregnancy within the year. Despite this, rates of modern contraceptive usage varied widely and unmet need ranged from 25% to 96%. Even amongst users of modern contraceptives, the uptake of the most effective long-acting reversible contraceptives (intrauterine devices) was low. Women of age less than 20 years, parity of two or less, limited education and those who deliver at home were at highest risk for having unmet need.

**Conclusions:**

Six weeks postpartum, almost all women wish to delay or prevent a future pregnancy. Even in sites where early contraceptive adoption is common, there is substantial unmet need for family planning. This is consistently highest amongst women below the age of 20 years. Interventions aimed at increasing the adoption of effective contraceptive methods are urgently needed in the majority of sites in order to reduce unmet need and to improve both maternal and infant outcomes, especially amongst young women.

**Study registration:**

Clinicaltrials.gov (ID# NCT01073475)

## Background

Demographic and Health Surveys (DHS) in 27 developing countries conducted between 1993 and 1996 have demonstrated that during the extended post-partum period, up to a year after delivery, most women wished to delay the subsequent pregnancy for two or three years or to prevent any future pregnancies altogether [1].

There are a number of safe and effective contraceptive methods that women can begin at various points after delivery, including those used immediately postpartum, to optimize birth spacing [2]. The provision of quality family planning services in the postpartum period has the potential to reduce the voluntary termination of unwanted pregnancies and effect a reduction in both maternal and childhood mortality and morbidity arising from unsafe abortions and inadequate spacing of births, respectively [3,4].

Short inter-pregnancy intervals are associated with an increased risk of low birth weight [5-7], preterm birth [8-10], small-for-gestational-age [11], as well as neonatal [12,13] and infant mortality [14]. The risk of adverse health outcomes is highest with a birth-to-pregnancy interval of less than six months [8,15]. Children born three to four years after a previous birth are likely to have a significant survival advantage compared to children born within two years of the previous birth [4,16,17]. Additionally, an early second pregnancy may negatively influence the health, development and survival of the first child [18].

Taking into consideration the demonstrated need for family planning postpartum and the potential for improving both maternal and child outcomes through effective birth spacing, there is a clear need to integrate postpartum contraception into maternal child health programs [19]; however, implementation of integrated programs remains limited [20].

Counseling for family planning during the antenatal period, considered the standard of care, is only offered to a fraction of women in developing countries, where few receive effective antenatal care [21]. Similarly, postpartum family planning counseling is infrequently provided [22]. In settings where home deliveries are common and postnatal care unlikely, there are few opportunities for postpartum contraception counseling [23]. Moreover, national family planning programs of many developing countries often neglect the needs of recently delivered women [23].

DHS data regarding contraceptive usage during the extended post-partum period from 17 countries between 2003 and 2007 demonstrated rates of unmet need ranging from 50% in Bangladesh to 88% in Mali. In this sample, women were likely to delay adoption of contraception to nine months after delivery. Postpartum women are more likely to have an unmet need for family planning than married women in general [24].

Even women who adopt modern contraceptive methods for birth spacing after delivery are likely to opt for short-term hormonal methods (injectable/oral contraceptives) [25]. In developing countries, many couples have difficulty using these methods correctly or consistently, which may lead to unintended pregnancies [26-28]. Long-acting reversible contraceptives (LARC) such as intrauterine devices (IUDs) are likely to be the most effective method for prevention of unwanted pregnancies, especially among those women who wish to use a contraceptive device to delay the next pregnancy [29].

The Statement for Collective Action for Postpartum Family Planning [19] and available research [1,24] focus on the extended postpartum period. However, fertility may return as early as 25 days after delivery [30]. While breastfeeding delays the onset of fertility significantly, [31] even lactating women may ovulate prior to the first menses, limiting their ability to accurately predict a return to fecundity [32]. It has been suggested that women who wish to prevent or delay a subsequent pregnancy after delivery should adopt a contraceptive method as early as possible after delivery and before resumption of sexual activity [24].

This study aims to assess the fertility intention, contraceptive usage and mix, as well as the unmet need for family planning amongst women who are six weeks postpartum using the Global Network for Women and Children’s Health Research’s Maternal and Newborn Health Registry (MNHR), a prospective multicentre active surveillance mechanism on-going in rural communities in five low and lower middle-income countries [33]. We also aim to assess which women are at greatest risk of having an unmet need for family planning during this period.

## Methods

### Study design, setting and participants

We used data collected by the NICHD’s Global Network for Women and Children’s Health Research Maternal and Newborn Health Registry (MNHR), a multi-site, prospective, ongoing, active surveillance system to track pregnancies and births in specific rural communities in Guatemala, India (2 states), Kenya, Pakistan and Zambia. Through the Registry, all pregnant women who are residents of these rural geographic clusters are recruited and followed to delivery and in the post-partum period. Information about the health of the mother and infant during the antenatal, labor and delivery and postnatal period is collected. Each cluster has a minimum of 300 deliveries per year and data from all consenting pregnant women are included in the Registry database. The Registry is described in detail elsewhere [33].

In brief, information on the eligible pregnant women and their babies is obtained at three time points. The first visit, at enrollment, ideally occurs by week 20 of gestation and information on the date of last menstrual period, estimated delivery date, age, level of schooling, parity, and status of last child born is collected. The second visit occurs within 48 hours of delivery and information collected includes prenatal care, birth preparedness, complications occurring during pregnancy, details of labor and delivery, including place, mode of delivery, provider and practices birth weight, status of the mother and newborn following delivery, referrals, and treatment provided to the mother and newborn at referral facilities. Interval maternal and newborn health and vital status is assessed at a third visit on day 42 after birth.

The study was approved by all of the involved institutions’ ethics review committees including the committees in the US institutions that partnered with each of the foreign sites. The study is registered at clinicaltrials.gov (NCT01073475). A Data Monitoring Committee appointed by NICHD reviews the Registry data on at least an annual basis. All women provide consent to participate in the Registry study.

For this study, questions adapted from the Demographic and Health Survey were added to the MNHR Questionnaire to assess fertility intention and contraceptive usage at day 42 post-partum [26]. Data were collected between April 2011 and September 2012 in all Global Network sites with specific start and end dates varying by site. Belgaum’s time for data collection was the shortest, explaining the smaller number of subjects at that site. All study data were obtained by trained interviewers who recorded the responses on case report forms.

### Study variables

Fertility intention was assessed through questions as to whether the woman would like to have another child or not and when she would like to have her next child. Specifically, women were asked about their future plans for child bearing. Those who professed wanting more children or those who were undecided were asked how long they would like to wait before the birth of the next child. Women were classified as those who wanted to prevent any future pregnancies, those who wanted more children, and those with ambivalent fertility intentions [34].

In order to assess the use of modern contraceptive methods, women were asked: “Are you currently doing something or using any method to delay or avoid getting pregnant?” If yes, they were asked which method they were currently using. Modern methods included pills, injections, condoms, IUDs, and female and male sterilization. The definition of usage of contraceptive methods was limited to modern methods because of the high failure rates of traditional methods.

Women with an unmet need for family planning were defined as those who have had a recent delivery, thus presumed to be fecund, and report not wanting any more children at all or wanting to delay the birth of their next child; but not using any method of contraception. Women with ambivalent fertility intentions were excluded from the definition of unmet need; giving us a conservative definition of unmet need.

Covariates included maternal age, literacy, parity, last inter-pregnancy interval, vital status of the last delivery, sex of the last delivered child, and number of currently living children, as well as the nature of antenatal, delivery and postnatal care received.

### Analysis

Data were entered at each study site, where data edits were performed prior to transmission to a central data center (RTI International, Durham, NC) where additional data edits were performed. Data were analyzed centrally and statistical analyses performed using SAS v. 9.3. Descriptive analyses were performed. Relative risks were obtained from a Poisson regression model adjusting for site and a single demographic characteristic with general estimating equations adjusting for cluster. Next, the demographic characteristics, which were statistically significant individually at p<0.1, were included in a multi-variable regression model. For this model, relative risks were obtained from a multi-variable Poisson regression model adjusting for site, maternal age, maternal education, parity, delivery location, last birth outcome and infant gender with general estimating equations adjusting for cluster.

## Results

A total of 36,687 women in the post-partum period were included in this study with the largest number (n=13,460) from Thatta, Pakistan (Table 1). Less than 5% (1,705/36,687) were desirous of another pregnancy immediately or within months of the index delivery. Variation between sites in fertility intention with rates of women wishing to delay or prevent a future pregnancy ranged from 93% in the Belgaum, India site to 99.7% in the Chimaltenango, Guatemala site. Despite the intent to delay or prevent a future pregnancy, usage of modern contraceptive methods at 42 days post-partum varied widely between sites and ranged from 73.5% in Kafue, Zambia and 65.5% Nagpur, India to 4% in Thatta, Pakistan.

**Table 1:** Post-partum contraceptive use and reproductive intention in sites of the Global Network 2011 - 2013

Country	Guatemala	Zambia	Kenya	Pakistan	India	
Site	Chimaltenango	Kafue	Western Kenya	Thatta	Belgaum	Nagpur

Clusters, n	10	10	16	24	20	20

Forms completed, n	4,460	1,618	7,140	13,460	1,679	8,330

Unmet need, n (%)						

Total	2,042 (67.6)	323 (25.5)	2,695 (46.0)	10,055 (96.6)	871 (64.0)	2,307 (31.6)

To limit pregnancies	540 (58.3)	35 (22.4)	388 (36.1)	1,639 (92.4)	190 (41.9)	733 (23.5)

To space pregnancies	1,502 (71.7)	288 (25.9)	2,307 (48.2)	8,416 (97.4)	681 (75.0)	1,574 (37.6)

Contraceptive use, n (%)						

Total	1,150 (25.8)	1,189 (73.7)	3,645 (51.1)	540 (4.0)	549 (32.7)	5,465 (65.6)

To limit	387 (41.7)	121 (77.6)	688 (63.9)	134 (7.6)	263 (58.1)	2,384 (76.5)

To space	602 (27.2)	866 (73.5)	2,533 (49.5)	233 (2.4)	238 (22.8)	2,767 (59.5)

The types of contraceptives also varied by site (Table 2). Permanent methods were used most frequently in the Asian sites. Long-acting, reversible contraceptives were used by more than 10% of subjects in the two Indian sites, and 3% or less in the other sites. Short-term contraceptive methods (oral or injectable hormonal contraceptives or condoms) were used by more than 90% of those using modern contraception in the African sites.

**Table 2:** Contraceptive mix among women using a modern contraceptive method across Global Network sites, 2011-2013

Country	Guatemala	Zambia	Kenya	Pakistan	India	
Site	Chimaltenango	Kafue	Western Kenya	Thatta	Belgaum	Nagpur

Any modern contraceptive method, n	1,150	1,189	3,645	540	549	5,465

Injectable hormonal contraceptives, n (%)	721 (63%)	469 (39%)	2,171 (60%)	131 (24%)	1 (0%)	1 (0%)

Birth control pills, n (%)	43 (4%)	463 (39%)	897 (25%)	69 (13%)	76 (14%)	276 (5%)

Condoms, n (%)	39 (3%)	234 (20%)	266 (7%)	96 (18%)	124 (23%)	2,637 (48%)

Intrauterine device, n (%)	8 (1%)	40 (3.4%)	86 (2.4)	11 (2%)	80 (14.6)	687 (12.6%)

Vasectomy, n (%)	2 (0%)	0 (0%)	0 (0%)	8 (2%)	1 (0%)	88 (2%)

Tubal ligation, n (%)	160 (14%)	10 (1%)	108 (3%)	166 (31%)	244 (44%)	1,771 (34%)

Other method, n (%)	177 (15%)	54 (5%)	148 (4%)	17 (3%)	8 (2%)	6 (0%)

## Unmet need for family planning

**Figure 1:**
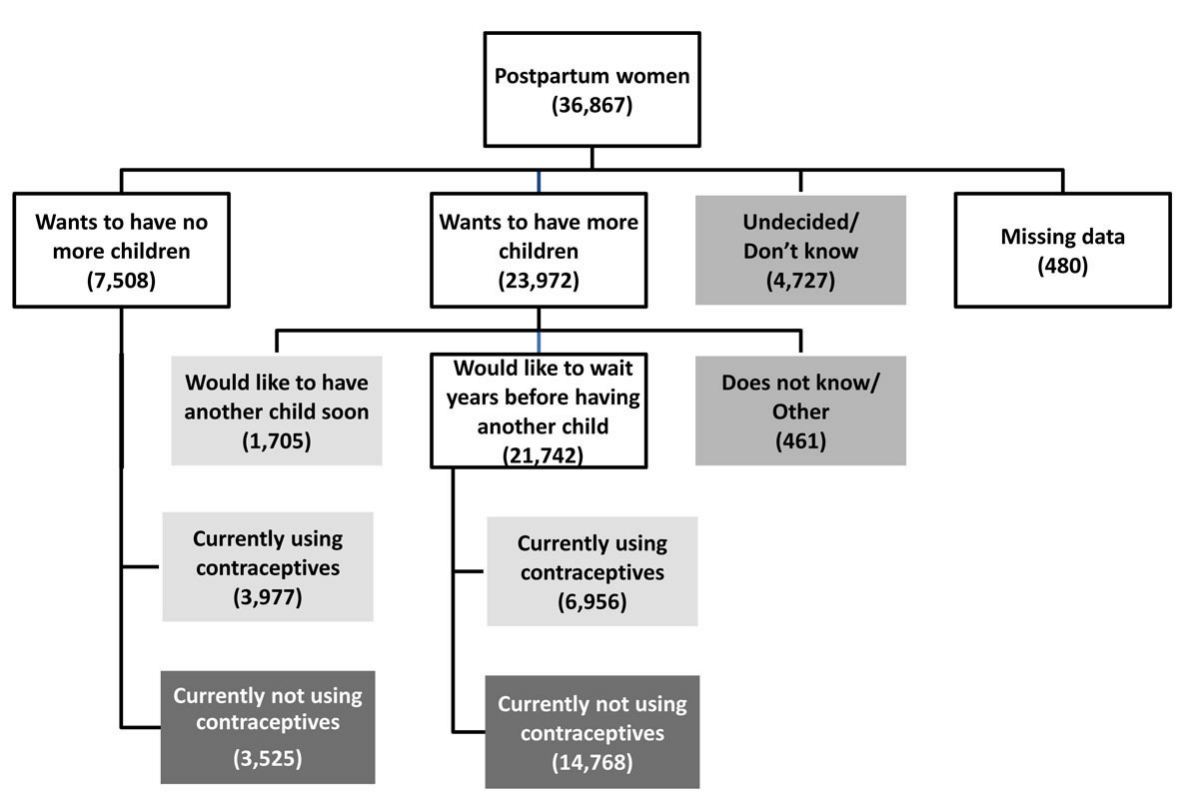
Fertility intentions, contraceptive usage and unmet need for family planning across the Global Network sites

Amongst women who wished to delay or prevent a future pregnancy, 50% had an unmet need for family planning services. One in five women expressed a desire to prevent all future pregnancies. Almost half of these women were not using a modern contraceptive at the time of the interview. Of the 65% of women who expressed a desire for more children, 91% wished to delay their next pregnancy by at least a year. Amongst this group, 70% were not using a modern contraceptive method. Of the women who were undecided about whether they want another child or not, 75% were not using a modern contraceptive method (Figure 1).

In Zambia, the overall contraceptive usage rate was 74%; however, 22% of women seeking to prevent birth and 26% of women wanting to delay their next pregnancy had an unmet need for family planning. Similarly, in Nagpur, India, 23% of women who wanted no more pregnancies at all and 38% of women who wanted to delay their next pregnancy by at least a year had an unmet need for family planning. In Kenya 36% of women had an unmet need for family planning to prevent births and 48% an unmet need for birth spacing. In the sites with lower rates of contraceptive usage, the unmet need was even higher (Table 1).

The women at greatest risk for having an unmet need for family planning were young women below the age of 20 years. Compared to women over the age of 30, these young mothers were more likely to report wanting to limit or delay future pregnancies whilst not using any family planning method (aRR=1.24; 95% CI: 1.16-1.33). In our data, unmet need is slightly higher amongst women with a parity of two or less (aRR=1.05; 95% CI: 1.02-1.08), woman with no formal education (aRR=1.05; 95% CI: 1.02-1.07) and women who deliver at home (aRR=1.05; 95% CI: 1.01-1.10). None of the other variables in the model, including outcome of last birth, gender of recently delivered child, or access to antenatal care were significantly related to unmet need for family planning. (Table 3)

**Table 3:** Unmet Need for Family Planning in the Global Network sites 2011 - 2013

Characteristic	Unmet Need for Family Planning N (%)	Met Need for Family Planning N (%)	Relative Risk^1^ Adjusted by Site (95% CI)	Multi-variable Analysis Adjusted Relative Risk^2^(95% CI)
Maternal age (years)				

< 20	1,859 (10.8)	970 (9.3)	1.28 (1.18, 1.39)	1.24 (1.16, 1.33)

20-29	11,381 (66.1)	8,056 (77.1)	1.05 (1.02, 1.08)	1.02 (1.00, 1.04)

≥ 30	3,965 (23.0)	1,429 (13.7)	--	--

Maternal education				

No formal education	8,500 (49.4)	799 (7.6)	1.04 (1.01, 1.07)	1.05 (1.02, 1.07)

Any formal education	8,705 (50.6)	9,656 (92.4)	--	--

Parity				

0-2	11,606 (67.5)	8,329 (79.7)	1.09 (1.04, 1.14)	1.05 (1.02, 1.08)

≥ 3	5,599 (32.5)	2,126 (20.3)	--	--

Antenatal care				

Any	15,322 (89.1)	10,329 (98.8)	--	

None	1,883 (10.9)	126 (1.2)	1.01 (0.98, 1.04)	

Delivery location				

Hospital	5,683 (33.0)	4,540 (43.4)	--	--

Clinic	3,991 (23.2)	3,138 (30.0)	1.01 (0.98, 1.05)	1.02 (0.98, 1.05)

Home/Other	7,531 (43.8)	2,777 (26.6)	1.05 (1.00, 1.10)	1.05 (1.01, 1.10)

Infant gender				

Male	8,826 (51.3)	5,445 (52.1)	--	--

Female	8,379 (48.7)	5,010 (47.9)	1.02 (1.00, 1.03)	1.02 (1.00, 1.03)

Neonatal status				

Baby alive until 6 weeks	16,219 (94.3)	10,215 (97.7)	--	

Neonatal mortality	507 (2.9)	125 (1.2)	1.05 (0.99, 1.10)	

Stillbirth	479 (2.8)	115 (1.1)	1.02 (0.97, 1.07)	

^1^ Relative Risks obtained from a Poisson regression model adjusting for site and a single demographic characteristic with general estimating equations adjusting for cluster.

^2^ Relative Risks obtained from a multivariate Poisson regression model adjusting for site, maternal age, maternal education, parity, delivery location and infant gender with general estimating equations adjusting for cluster.

## Discussion

The six sites of the Global Network included in this study span five low and low-middle income countries across three continents, having unique geographic, social, religious and contextual characteristics. Despite these differences, at six weeks postpartum, almost all women are consistent in their desire to delay or prevent a future pregnancy. This constancy of pregnancy intention does not correlate with actual usage of a modern contraceptive method, which varies substantially between the sites from a low of 4% in Thatta, Pakistan to a high of 73.5% in Kafue, Zambia.

Despite this difference in contraceptive usage rates, across all sites, there is a clear link between occurrence of unmet need for family planning and young age. Women below the age of 20 years are most likely to have unmet need for family planning. This is particularly concerning as pregnancy and childbirth are the leading causes of death among females between 15 to 19 years of death [35]. These data suggest that postpartum family planning programs need to prioritize provision of care to young mothers, for whom delaying a subsequent pregnancy could be potentially life-saving.

The high rates of early postpartum contraceptive usage in two of the Network’s six sites, specifically Kafue, Zambia, and Nagpur, India are encouraging. This suggests that it is possible for women in the postpartum period to translate their desire to delay or prevent a pregnancy into practice. However, this success is tempered as almost all women not using a contraceptive method in both of these sites have an expressed preference for delaying or preventing future pregnancies.

Even amongst women using a modern contraceptive method in most sites, including Kafue, Zambia, the rate of uptake of the most effective methods for reliably spacing births, i.e. long-acting reversible contraceptives is very low. The exceptions are seen in both Indian sites. As demonstrated in the site at Nagpur, India, it is possible to raise overall contraceptive usage and to increase uptake of intrauterine devices within six weeks of delivery. Delivery at a health care facility presents an ideal opportunity for IUD insertion. Immediate post-partum insertion of intrauterine devices is safe and effective, though expulsion rates appear to be higher than with interval insertion. As demonstrated by the data from the Global Network sites, motivation to delay or prevent future pregnancies is high during this period [36]. In sites such as Nagpur, where postnatal follow-up occurs more reliably, interval insertion, i.e. at six weeks postpartum may be a reasonable option.

The very low usage of contraceptive methods in Thatta, Pakistan is a cause of concern. In fact, this is much lower than the overall rate of contraceptive use in the country as measured by the Pakistan Demographic and Health Survey (PDHS) 2013, which estimates that 23.1% of women resident in rural areas use a modern family planning method [37]. This may be reflective of local variation, as the data for this paper are restricted to a single rural district in Sindh province. Conversely, it may indicate that uptake of modern contraceptive methods is particularly poor early in the postpartum period. According to PDHS data, two months after delivery, most women in Pakistan have resumed sexual activity. Thus the need for early postpartum adoption of contraception is particularly important for prevention of unwanted or mistimed pregnancy.

The persistence of unmet need for family planning, particularly the high rate of unmet need amongst women who express a preference for more children but wish to delay future pregnancies, points to an important gap in service provision, particularly in those sites with low contraceptive usage rates.

At six weeks postpartum, when the data for this study were collected, approximately 90% of women who receive counseling regarding exclusive breastfeeding, practice it (data not shown). One of the weaknesses in this study is the lack of information on the effective use of lactational amenorrhea (LAM). We do not know what proportion of women in the study view their breastfeeding as a form of contraception, thus delaying adoption of a modern family planning method. However, data from other developing countries shows that only 26 percent of reported LAM users meet the criteria for correct LAM practice [38]. Thus, while reported exclusive breastfeeding rates are high, the number of women protected from unwanted pregnancy through this practice is unknown.

We report the results from specific sites in the Global Network countries, which may not be reflective of the each country as a whole. However, the consistency of women’s expressed wish to delay or limit future pregnancies at 6 weeks postpartum points to the importance of early provision of family planning services. Similarly, the findings regarding high rates of unmet need, which are consistent across all sites, point to the need for further research for interventions to improve access to family planning services for this group.

## Competing interests

The authors declare that they have no conflicts of interest.

## Author contributions

OP developed the study protocol with input from SSG, AP, AG, FE, EC, BK, SS, RJD, EAL, PLH, KMH, NFK, WAC, EMM, MKT, and RLG. OP, SSG, AP, AG, FE, EC, BK, SS oversaw the study implementation at each site. JLM performed the analyses with input from OP and EMM. OP and RLG wrote the initial manuscript; all authors reviewed the final manuscript.

## Peer review

Reviewer reports for this article can be found in Additional file 1.

## Supplementary Material

Additional file 1 Click here for file

## Declarations

This article has been published as part of *Reproductive Health* Volume 12 Supplement 2, 2015: Research reports from the NICHD Global Network for Women’s and Children’s Health Research Maternal and Newborn Health Registry. The full contents of the supplement are available online at http://www.reproductive-health-journal.com/supplements/12/S2. Publication of this supplement was supported by grants from the Eunice Kennedy Shriver National Institute of Child Health and Human Development to RTI International.
