# Supplementary material for: Postpartum contraceptive use and unmet need for family planning in five low-income countries
Source: Reprod Health. 2015 Jun 8;12(Suppl 2):S11. doi: 10.1186/1742-4755-12-S2-S11 (PMC4464604; doi:10.1186/1742-4755-12-S2-S11)
Supplement: Additional file 1 [file 1742-4755-12-S2-S11-S1.pdf]

Referee's comments to the authors– this sheet WILL be seen by the author(s) and published with the article

Title	Postpartum Contraceptive Use and Unmet Need for Family Planning in 5 low-income countries
Author(s)	Omrana Pasha, Shivaprasad S Goudar, Archana Patel, Ana Garces, Fabian Esamai, Elwyn Chomba, Janet Moore, Bhalchandra Kodkany, Sarah Saleem, Richard J Derman, Edward A Liechty, Patricia L Hibberd, K Michael Hambidge, Nancy F Krebs, Waldemar A Carlo, Elizabeth M McClure, Marion Koso-Thomas, Robert L Goldenberg
Referee's name	Lamar Ekbladh

When assessing the work, please consider the following points, where applicable:

1. **Is the question posed by the authors new and well defined?**
2. **Are the methods appropriate and well described, and are sufficient details provided to replicate the work?**
3. **Are the data sound and well controlled?**
4. **Does the manuscript adhere to the relevant standards for reporting and data deposition?**
5. **Are the discussion and conclusions well balanced and adequately supported by the data?**
6. **Do the title and abstract accurately convey what has been found?**
7. **Is the writing acceptable?**

Please make your report as constructive and detailed as possible in your comments so that authors have the opportunity to overcome any serious deficiencies that you find and please also divide your comments into the following categories:

- Major Compulsory Revisions (which the author must respond to before a decision on publication can be reached)
- Minor Essential Revisions (such as missing labels on figures, or the wrong use of a term, which the author can be trusted to correct)
- Discretionary Revisions (which are recommendations for improvement but which the author can choose to ignore)

Where possible please supply references to substantiate your comments.

When referring to the manuscript please provide specific page and paragraph citations where appropriate.

General comments: This is an excellent report of over 36,000 patients from 6 sites in 5 countries. over ⇒

Major compulsory revisions: NONE

Minor essential revisions: NONE

Discretionary revisions: I would have liked to see some ~~explanation~~ of the discrepancy between sites (and) discussion

(continue on the next sheet)

Continued:

General comments: The results are not suprising in general, but the fact that 90+% of women at 42 days post partum desire to delay or prevent pregnancy is impressive. The results are well presented and the discussion appropriate.

Discretionary: relative to why (social, economic, education religious, availability) and since use of LARC was considered important, how the woman's desires might be better met. Also, at what points in each site might the issues be best addressed.

Referee's comments to the authors– this sheet <u>WILL</u> be seen by the author(s) and published with the article

Title	Postpartum Contraceptive Use and Unmet Need for Family Planning in 5 low-income countries
Author(s)	Omrana Pasha, Shivaprasad S Goudar, Archana Patel, Ana Garces, Fabian Esamai, Elwyn Chomba, Janet Moore, Bhalchandra Kodkany, Sarah Saleem, Richard J Derman, Edward A Liechty, Patricia L Hibberd, K Michael Hambidge, Nancy F Krebs, Waldemar A Carlo, Elizabeth M McClure, Marion Koso-Thomas, Robert L Goldenberg
Referee's name	Russell Kirby

When assessing the work, please consider the following points, where applicable:

1. Is the question posed by the authors new and well defined?
2. Are the methods appropriate and well described, and are sufficient details provided to replicate the work?
3. Are the data sound and well controlled?
4. Does the manuscript adhere to the relevant standards for reporting and data deposition?
5. Are the discussion and conclusions well balanced and adequately supported by the data?
6. Do the title and abstract accurately convey what has been found?
7. Is the writing acceptable?

Please make your report as constructive and detailed as possible in your comments so that authors have the opportunity to overcome any serious deficiencies that you find and please also divide your comments into the following categories:

- Major Compulsory Revisions (which the author must respond to before a decision on publication can be reached)
- Minor Essential Revisions (such as missing labels on figures, or the wrong use of a term, which the author can be trusted to correct)
- Discretionary Revisions (which are recommendations for improvement but which the author can choose to ignore)

Where possible please supply references to substantiate your comments.

When referring to the manuscript please provide specific page and paragraph citations where appropriate.

General comments:
This paper is nicely done and makes a useful contribution to the literature. This review did note a few areas for potential revision.

Major compulsory revisions:
Comments provided under minor revisions below.

Minor essential revisions:
P 4 second para – this reviewer is a highly experienced perinatal epidemiologist, but has not previously encountered the term 'late postpartum period' as defined here or otherwise. Provide a reference from the peer-reviewed literature for this usage, or explain more fully why this term should be used.

P 6 under analysis – why was p<0.08 selected as the cutoff? This is unconventional, Hosmer and Lemeshow recommend p<0.20 as a conservative cutoff, other researchers use p<0.10.

(continue on the next sheet)

Continued:

Same paragraph – the Poisson regression model was ‘multivariable’ not multivariate. The previous sentence uses the correct term.

Last line p 8 onto p 9, reword the sentence so its not necessary to spell out 1705 in words.

As a general comment, the text in results on p 9-10 describing descriptive results seems overly long, and could probably be focused, since the data referred to are also provided in the tables. This review is happy to leave a final decision on that to the journal editors. However, if the overall length of the manuscript is deemed appropriate, it seems unbalanced to this reviewer to devote only a paragraph on p 11 to presentation of the multivariable adjusted results, which are the crux of the paper, while devoting some two pages to descriptive statistics for the various study sites.

There are some other interesting results that could be mentioned in the text, but no inferential results are included, just the figure 25% not expressed as an adjusted odds ratio and without a confidence interval.

In Table 1, if space permits, consider adding a word after ‘limit’ and ‘space’ in the first column to make these statistics more understandable to readers not already familiar with this work.

Figure 1 is very nice with bright colors, but it would be best to use grey-tones instead as many readers in resource poor countries will be unable to read the figure when printed in black and white.

Table 3, last column, change ‘Multivariate’ to ‘Multivariable’, also in note 2 to this table.
